# Co(II)-Based Metal-Organic Framework Derived CA-CoNiMn-CLDHs with Peroxidase-like Activity for Colorimetric Detection of Phenol

**DOI:** 10.3390/ma16186212

**Published:** 2023-09-14

**Authors:** Wenjie Tan, Rui Xin, Jiarui Zhang, Lilin Yang, Min Jing, Fukun Ma, Jie Yang

**Affiliations:** 1 School of Material Science and Engineering, Shandong Jianzhu University, Jinan 250022, Chinazhangjiarui326@163.com (J.Z.);; 2 Shandong Jiazihu New Material Technology Co., Ltd., Jinan 250022, China; 3 Department of Pharmaceutical and Bioengineering, Zibo Vocational Institute, Zibo 255000, China

**Keywords:** metal organic framework, nanozyme, colorimetric detection, phenol

## Abstract

Given the serious harm of toxic phenol to human health and the ecological environment, it is urgent to develop an efficient, low-cost and sensitive nanoenzyme-based method to monitor phenol. MOF-derived nanozyme has attracted wide interest due to its hollow polyhedra structure and porous micro-nano frameworks. However, it is still a great challenge to synthesize MOF-derived multimetal synergistic catalytic nanoenzymes in large quantities with low cost. Herein, we reported the synthetic strategy of porous hollow CA-CoNiMn-CLDHs with ZIF-67 as templates through a facile solvothermal reaction. The prepared trimetallic catalyst exhibits excellent peroxidase-like activity to trigger the oxidative coupling reaction of 4-AAP and phenol in the presence of H_2_O_2_. The visual detection platform for phenol based on CA-CoNiMn-CLDHs is constructed, and satisfactory results are obtained. The K_m_ value for CA-CoNiMn-CLDHs (0.21 mM) is lower than that of HRP (0.43 mM) with TMB as the chromogenic substrate. Because of the synergistic effect of peroxidase-like activity and citric acid functionalization, the built colorimetric sensor displayed a good linear response to phenol from 1 to 100 μM with a detection limit of 0.163 μM (3σ/slope). Additionally, the CA-CoNiMn-CLDHs-based visual detection platform possesses high-chemical stability and excellent reusability, which can greatly improve economic benefits in practical applications.

## 1. Introduction

Phenol is a common water contaminant, mainly from the plastics, dyes, chemical plants and pharmaceutical industry. It is one of cumulative environmental contaminants that threaten human health and the ecosystem due to the high toxicity, carcinogenicity and difficulty of removal in natural conditions [1,2,3]. Phenol has acute and chronic toxicity to humans and other organisms, even at low concentrations (<10 mg/L). Human contact with phenol over the long term will cause abnormal breathing, coma and death, while the high concentration in water will lead directly to aquatic organisms’ death [4,5,6]. Phenol has been identified by the US Environment Protection Agency (USEPA) as a major contaminant [7,8]. Therefore, the accurate and sensitive detection of phenol in water is a vital factor for effective remediation of phenol polluted water bodies.

As research continues, various detection techniques have been developed for the detection of phenol, including chromatography, electrochemistry and spectrophotometry [9,10,11]. Among them, electrochemical biosensor can be employed to test the electrooxidation of phenol in acidic and alkaline solutions by cyclic voltammetry or square wave voltammetry, thus realizing the sensitive determination for phenol [12,13]. Additionally, high-performance liquid chromatography (HPLC) is a highly selective and sensitive analytical method, while the complex matrices and trace phenol in real samples usually require separation and pre-concentration steps [14]. The above analytical methods can obtain good measuring results, and the detection limit even reaches the nanomolar scale. However, they usually require sophisticated instruments, a complex pre-treatment process, long analysis time and trained personnel, which can become troublesome when detecting on the field. Thus, developing a facile, low-cost and efficient sensor for the detection of trace phenol is essential and appealing. In this regard, the enzymatic colorimetric assay is an alternative to overcome these constraints for phenol identification, because of its operational simplicity, time efficiency, low cost and naked-eye visualization [15,16,17]. Traditionally, the enzymatic colorimetric assay needs the participation of natural enzymes, such as the horseradish peroxidase enzyme (HRP). However, the HRP can be easily deactivated, and has a higher cost and difficult storage limit due to its large-scale application [18]. Therefore, various artificial enzymes have been developed to surmount the shortcomings of natural enzymes and expand the applications of the colorimetric assay.

Nanozymes, which are nanomaterials with enzyme-like activities, can catalyze enzyme substrates into products under the right catalytic reaction conditions following enzyme kinetics. Compared to natural enzymes, nanozymes exhibit the significant properties of simple preparation, high stability, low cost, high-catalytic performance and long-term storage, which have been successfully synthesized and widely used in biosensors [17,19]. In particular, various inorganic nanozymes with peroxidase-like catalytic activity have been successfully used in the colorimetric detection of contaminants, including carbon-based materials [20], noble metals [21], metal oxide [22], metal-organic frameworks (MOFs), and derivatives [23]. Among them, MOFs are a hybrid nanomaterial formed through strong coordination bonds between metal ions and organic ligands, which have attracted wide attention due to their significant advantages of their high specific surface area, abundant pores, open active sites and ordered crystalline structures, etc. [24,25,26]. Therefore, MOFs and their derivatives with peroxidase-like activity are applied for environmental remediation [27]. Li et al. fabricated a MOF-derived Co-based nanozyme for biosensing and efficient degradation of organic pollutants [28]. Chen et al. reported a MOF-derived oxide double-shelled nanozyme with efficient peroxidase-like activity for biosensing and dye degradation [29]. However, the narrow micropore and poor hydrophilicity of MOFs are not conducive to the diffusion of substances, which hinders the effective contact between pollutants and active sites [30].

MOF-based nanozymes, with abundant mesoporous derived from MOFs as templates or precursors, are ideal artificial enzymes. Zeolitic imidazolate frame-work-67 (ZIF-67), with excellent catalytic performance, can be employed as an ideal template to fabricate 3D hollow-structured nanozymes [31]. In particular, ZIF-67-derived layered double hydroxides (LDHs) are a hierarchical hollow architecture with 2D nanosheets uniformly grown on the surface. The unique construction avoids excessive accumulation of LDH nanosheets and insufficient utilization of active sites [32]. After the high-temper calcination of LDHs (CLDHs), the metal ions in the main layer plate of LDHs are transformed into mixed metal oxides with a more stable structure, and the metal active sites introduced into the main layer through the lattice confinement are highly dispersed [33]. Therefore, the LDH-based nanozymes derived from ZIF-67 are the ideal candidate for artificial enzymes. Moreover, the combination of multimetal synergistic catalysis, surface functionalization and component proportion of nanomaterials can affect the catalytic activity of nanozymes [34,35]. Thus, if multi-metal active center (Co, Ni, Mn) LDHs derived from ZIF-67 are fabricated, it is anticipated that an ideal nanozyme with high-catalytic activity can be obtained.

Inspired by the above discussion, we synthesized a novel 3D hierarchical CA-CoNiMn-CLDHs through a facile fabrication process with outstanding peroxide-like activity for the colorimetric detection of trace phenol in water. The MOF-derived material system was designed by ingeniously integrating citric acid (CA)-modified and porous CoNiMn-CLDHs, and each component worked together to realize the visually sensitive detection of phenol. Specifically, small-sized ZIF-67 was employed as a template to synthesize the CoNiMn-CLDHs with hollow polyhedral structures. The ultrathin nanosheets grew vertically on the polyhedron, avoiding the aggregation of free CLHDs nanosheets as well as providing abundant mesopores, which facilitated the substrate molecular diffusion and exposed more active sites. The peroxidase-like activity of ZIF-67 derived tri-metal enzyme (CoNiMn-CLDHs) is superior to the bimetallic enzyme, and is enjoying significant cost and field operation advantages. On one hand, the use of expensive precious metals was avoided in the material system and replaced with cheap transition metals with the same performance. On the other hand, the established colorimetric array could be operated on site without expensive instruments and complicated operation. In addition, the citric acid (CA) modification and the strong synergy between Co, Ni and Mn ions also contribute to the improvement of enzyme-like catalytic activity. The CA-CoNiMn-CLDHs with peroxidase-like activity catalyzed the oxidative coupling between phenol and 4-aminoantipyrine (4-AAP) in the presence of H_2_O_2_ and determined the concentration of phenol by obvious color changes. The established colorimetric assay displayed excellent sensitivity and high selectivity, and the limit of detection (LOD) was 0.163 μM, which provided a reference for developing a high-performance sensor.

## 2. Experimental Section

### 2.1. Reagents and Materials

Co(NO_3_)_2_·6H_2_O, 2-methylimidazole (2-MIM), citric acid (CA), sodium azide (NaN_3_), p-benzoquinone (BQ), isopropanol (IPA), 4-aminoantipyrine (4-AAP) and 3,3′,5,5′-tetramethylbenzidine (TMB) were all obtained from Aladdin Reagent Co., Ltd. (Shanghai, China). Ni(NO_3_)_2_·6H_2_O and MnCl_2_·4H_2_O were provided by Shanghai Macklin Biochemical Technology Co., Ltd. (Shanghai, China). CH_3_COONa were purchased from Damao Chemical Reagent Factory (Tianjin, China). N,N-dimethyl formamide (DMF), ethanol (EtOH) and methanol were obtained from Sinopharm Chemical Reagent Co., Ltd. (Shanghai, China). H_2_O_2_ was received from Yantai Far East Fine Chemical Co., Ltd. (Yantai, China). All the chemicals used in this study were of analytical grade.

### 2.2. Characterization

The scanning electron microscopy (SEM) images were provided by field-emission scanning electron microscopy (ZEISS G500, Jena, Germany). X-ray diffraction (XRD) patterns were obtained via X-ray diffractometer (Bruker D8, Mannheim, Germany) and 2θ was scanned from 5° to 80°. Fourier transforms infrared (FT-IR) spectra was performed on FT-IR spectrometer (Thomas Nicolet 670, Waltham, MA, USA). Transmission electron microscopy (TEM) images were analyzed using a transmission electron microscope (JEM-2010, Nara, Japan). N_2_ adsorption–desorption isotherm was measured by a physisorption analyzer (ASAP2020M+C, Micrometrics, Norcross, GA, USA) and the pore size distribution was estimated by the Barrett–Joyner–Halenda (BJH) method. The X-ray photoelectron spectroscopy (XPS) was identified using an XPS spectrometer (ESCALAB Xi+, Cambridge, UK). UV spectroscopy measurements were recorded by a UV-Vis spectrophotometer (HITACHI U-4100, Tokyo, Japan). The electron paramagnetic resonance (EPR) spectra were tested by an EPR spectrometer (Bruker EMXnano, Mannheim, Germany).

### 2.3. Synthesis

#### 2.3.1. Synthesis of ZIF-67 Templates

Firstly, Co(NO_3_)_2_·6H_2_O (582 mg) and 2-MIM (656 mg) were dissolved in 50 mL methanol and gently stirred at room temperature to form the uniform solutions, respectively. Subsequently, the Co(NO_3_)_2_·6H_2_O solution was slowly added to the 2-MIM solution and stirred uniformly, and then aged at room temperature for 24 h. After the reaction finished, the precipitate was centrifuged and washed with methanol 3–5 times, and finally dried at 60 °C overnight.

#### 2.3.2. Synthesis of CA-CoNiMn-CLDHs

The as-prepared ZIF-67 as templates (80 mg) were dispersed into 20 mL DMF and 20 mL EtOH mixed solvent under magnetic stirring at room temperature for 10 min to form a homogeneous solution. Ni(NO_3_)_2_·6H_2_O (80 mg) and MnCl_2_·4H_2_O (80 mg) were dissolved in 10 mL deionized water (DIW) by magnetic stirring, and then rapidly poured into the above solution under a continuous stir. The mixed solution was transferred into Teflon-lined autoclave and reacted at 90 °C for 1 h. After the reaction finished, the formed CoNiMn-LDHs were collected by centrifugation, washed 3 times with deionized water, and dried in air at 60 °C for 6 h. The obtained CoNiMn-LDHs were placed in tube furnace and calcined at 350 °C for 2 h with a heating rate of 1 °C/min to obtain hollow CoNiMn-CLDHs.

A total of 100 mg of CoNiMn-CLDHs was dispersed into 50 mL CA solution (5 mM) under magnetic stirring at room temperature for 30 min. The final products were collected by filtration and washed 3 times with deionized water and dried at 60 °C for 12 h.

As a control, CoNi-LDHs and CoMn-LDHs samples were prepared by the same synthesis process as CoNiMn-LDHs, but the addition amount of Ni(NO_3_)_2_·6H_2_O and MnCl_2_·4H_2_O was 160 mg, respectively. The experimental details are shown in Table 1. The calcination process was the same as CoNiMn-CLDHs. The products obtained were denoted as CoNi-CLDHs and CoMn-CLDHs, respectively.

### 2.4. Peroxidase-like Catalytic Activity and Steady-State Kinetics of CA-CoNiMn-CLDHs

The peroxidase mimetic activity of CA-CoNiMn-CLDHs was evaluated via the following typical colorimetric experiments: 400 μL of TMB (5 mM), 300 μL of CA-CoNiMn-CLDHs (0.5 mg/mL) and 300 μL of H_2_O_2_ (50 mM) were added into acetate buffer (pH 4.0, 0.2 M) and the total volume of colorimetric system was 3 mL. The mixture was maintained at ambient temperature for 20 min to obtain the oxidized TMB. The absorbance at 652 nm of colorimetric system was recorded by UV-Vis spectrophotometer.

In addition, the kinetic experiments were carried out to further evaluate the peroxide-like activity of CA-CoNiMn-CLDHs in the colorimetric system. The different concentrations of TMB (1–10 mM) and H_2_O_2_ (10–100 mM) were added to the colorimetric system, respectively. UV-Vis absorbance at 652 nm was recorded and the kinetics parameters (K_m_ and V_max_) were obtained through the Lineweaver–Burk plots of the Michaelis–Menten equation.

### 2.5. Colorimetric Detection of Phenol

Based on the peroxidase-like activity of CA-CoNiMn-CLDHs, 4-AAP was used as the chromogenic substrate for colorimetric detection of phenol. The colorimetric experiments were performed as follows: 700 μL 4-AAP (10 mM), 150 μL H_2_O_2_ (50 mM), 150 μL CA-CoNiMn-CLDHs (0.5 mg/mL) and 900 μL phenol (5 mM) were added into 1.1 mL acetate buffer (pH 4.0, 0.2 M) and kept at room temperature for 20 min. The absorbance of the reaction solution at 525 nm was measured by the UV-Vis spectrophotometer.

To investigate the influences of reaction conditions on the chromogenic reaction, the effects of pH (3.0–8.0), temperature (25–55 °C), incubation time (5–30 min), 4-AAP concentration (1–15 mM), H_2_O_2_ concentration (10–150 mM), and CA-CoNiMn-CLDHs concentration (0.1–2.0 mg/mL) on the peroxidase-like activity of CA-CoNiMn-CLDHs were explored to obtain the optimal reaction conditions.

Under the optimal reaction conditions, the colorimetric platform for phenol was built. In detail, 700 μL 4-AAP (10 mM), 150 μL H_2_O_2_ (70 mM), 150 μL CA-CoNiMn-CLDHs (0.7 mg/mL) and 900 μL different concentrations of phenol were added into 1.1 mL acetate buffer (pH 5.0, 0.2 M). After incubation at ambient temperature for 20 min, the absorbance of the mixture at 525 nm was measured.

### 2.6. Stability and Reusability of CA-CoNiMn-CLDHs

The recyclability experiments were performed to evaluate the reproducibility of the synthesized CA-CoNiMn-CLDHs. After the colorimetric determination of phenol, the CA-CoNiMn-CLDHs were collected by centrifugation, washed with deionized water for 3 times and recycled for the next run. The cycling experiment was performed 5 times following the details of the colorimetric experiment in Section 2.5.

## 3. Results and Discussion

### 3.1. Synthesis and Characterization of CA-CoNiMn-CLDHs

**Figure 1:**
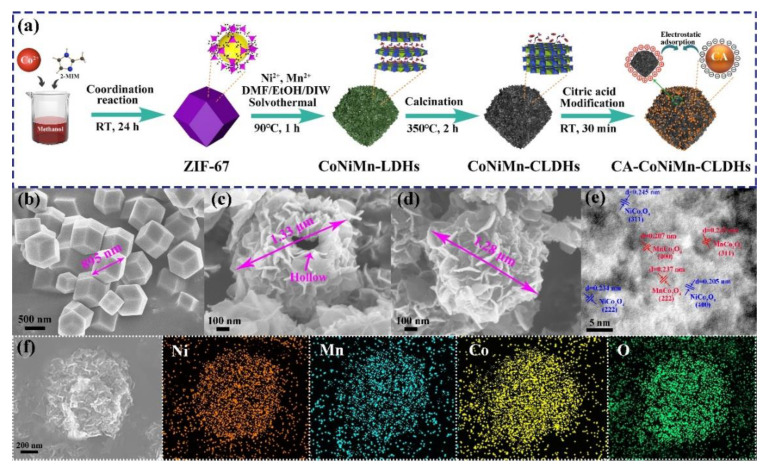
(**a**) Schematic illustration of the preparation of porous hollow CA-CoNiMn-CLDHs. SEM images of (**b**) ZIF-67, (**c**) CoNiMn-LDHs and (**d**) CA-CoNiMn-CLDHs. (**e**) HRTEM image of CA-CoNiMn-CLDHs. (**f**) EDS mapping of Ni, Mn, Co and O elements for CA-CoNiMn-CLDHs.

CA-CoNiMn-CLDHs were prepared under mild conditions through a facile step-by-step templating method using ZIF-67 as the template (Figure 1a). Firstly, Co^2+^ and 2-MIM were mixed together and coordinated at room temperature to form ZIF-67 of rhombic dodecahedron, which simultaneously acted as the template reactant and cobalt source. Subsequently, the ZIF-67-derived, hollow-structured CoNiMn-LDHs were prepared via the solvothermal method by adding Ni^2+^ and Mn^2+^ to the mixed solvent (DMF/EtOH) containing ZIF-67 templates. Ethanol with weak reducibility reacted with NO_3_^−^ to form hydroxide ions (OH^−^) during the solvothermal reaction [36]. The protons produced from the hydrolysis of Ni^2+^ and Mn^2+^ replaced the anions in 2-MIM, while partial Co^2+^ in ZIF-67 was oxidized to form Co^3+^ by NO_3_^−^ and O_2_; after that, Co^2+^/Co^3+^, Ni^2+^ and Mn^2+^ reacted with OH^−^ to form Ni(OH)_2_, Mn(OH)_2_, Co(OH)_2_ and Co(OH)_3_ monomers, which further grew freely via complexation reactions and eventually transformed into CoNiMn-LDHs with a rhomboidal dodecahedron structure by olation reaction and crystallization [36,37]. In addition, the mixed solvent (DMF/EtOH) provided an acidic environment for solvothermal reaction, owing to the generation of formate during the decomposition process, so that partial Co^2+^, Ni^2+^ and Mn^2+^ were oxidized to Co^3+^, Ni^3+^ and Mn^3+^ by NO_3_^−^. Simultaneously, the DMF and ethanol molecules in the mixed solvent could enter into the channels of hydroxide monomers and generate formate to cut off the original hydrogen bond network of LHDs, which reduced the transverse growth rate of LDHs nanosheets and formed great quantities of metastable hydroxyl groups on the surface of nanosheets. The metastable hydroxyl groups further induced the formation of coordinated unsaturated metal centers and reduced the thickness and packing density of nanosheets [38]. Finally, ZIF-67-derived, 3D hollow structures were assembled and obtained by interconnecting ultra-thin nanosheets. CoNiMn-LDHs further converted to CoNiMn-CLDHs after high-temperature calcination. After calcination, the negatively charged CA was modified on the positively charged surface of CoNiMn-CLDHs by electrostatic adsorption to enhance the peroxidase-like activity. Therefore, a colorimetric sensing platform based on the excellent nanozyme catalytic activity of CA-CoNiMn-CLDHs was designed for the visual detection of trace phenol in water.

The morphologies of the synthesized ZIF-67, CoNiMn-LDHs and CoNiMn-CLDHs were characterized by FESEM. As observed in Figure 1b, ZIF-67 presented regular dodecahedral morphology with a smooth surface, which possessed a uniform size of about 895 nm. Figure 1c displays the morphology of CoNiMn-LDHs, whose overall structure retained the regular decahedron shape of ZIF-67. The ultrathin nanosheets were vertically grown on the surface of the hollow polyhedron and crossed each other to form a dense shell. The thickness of nanosheets was measured to be about 10 nm. After high-temperature calcination, CoNiMn-CLDHs still maintained the porous rhombic dodecahedron structure, and the surfaces of polyhedron were composed of randomly oriented ultrathin nanosheets (Figure 1d). Additionally, Figure 1c,d clearly show the long and narrow pores formed by an edge-to-face stacking. The abundant pore structure greatly exposed active sites of the CoNiMn-CLDHs, and also improved the diffusion rate of substrate molecules, which was beneficial to enhance the detection sensitivity of pollutants. The crystal structure of CoNiMn-CLDHs was further characterized in detail by high-resolution TEM (HRTEM). As shown in Figure 1e, the CoNiMn-CLDHs with high crystallinity exhibited clear lattice fringes of NiCo_2_O_4_ and MnCo_2_O_4_. The lattice fringes of 0.245 nm, 0.234 nm and 0.205 nm corresponded to (311), (222) and (400) crystal planes of NiCo_2_O_4_. Additionally, the fringes corresponding to the lattice spacing of 0.249 nm, 0.237 nm and 0.207 nm could be assigned to the (311), (222) and (400) crystal planes of MnCo_2_O_4_. The HRTEM results revealed the successful formation of mixed metal oxide derived from CoNiMn-LDHs after calcination. Figure 1f shows the EDS mapping of CoNiMn-CLDHs, in which the Ni, Mn, Co and O elements were evenly distributed on the surface of polyhedron.

**Figure 2:**
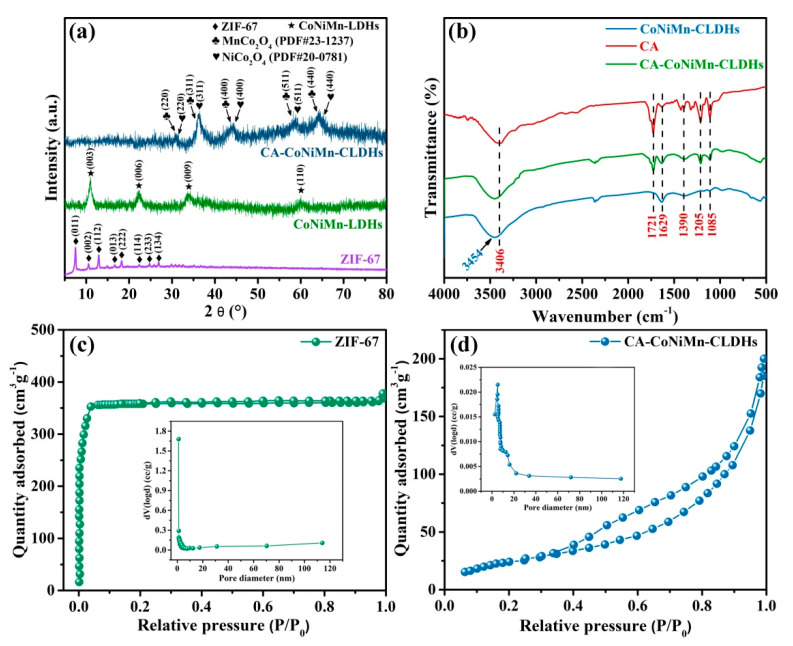
(**a**) XRD patterns of ZIF-67, CoNiMn-LDHs and CA-CoNiMn-CLDHs. (**b**) FT-IR spectra of CA, CoNiMn-CLDHs and CA-CoNiMn-CLDHs. N_2_-adsorption/sorption isotherms and pore size distribution (inset) of (**c**) ZIF-67 and (**d**) CA-CoNiMn-CLDHs.

The phase contents of ZIF-67, CoNiMn-LDHs and CA-CoNiMn-CLDHs were characterized by XRD patterns. In Figure 2a, a series of characteristic diffraction peaks of ZIF-67 at 7.53°, 10.64°, 12.91°, 16.69°, 18.29°, 22.36°, 24.81° and 26.88° agreed well with the (011), (002), (112), (013), (222), (114), (233) and (134) planes of the simulated ZIF-67 spectrum without other peaks observed, indicating that the high purity of ZIF-67 was successfully obtained [32]. The XRD pattern of CoNiMn-LDHs after the ion-exchange by Ni^2+^ and Mn^2+^ showed the characteristic peaks of a hydrotalcite-like compound. The diffraction peaks at 11.02°, 22.45°, 33.88° and 59.95° correspond to the (003), (006), (009) and (110) planes, respectively [39]. Meanwhile, the diffraction peaks of ZIF-67 disappeared, revealing that the crystal structure was completely changed. After calcination in the air, several diffraction peaks clearly appeared at 31.15°, 36.39°, 44.24°, 59.09° and 64.98° for CA-CoNiMn-CLDHs, which matched well with the (220), (311), (400), (511) and (440) planes of NiCo_2_O_4_ (PDF#20-0781). Additionally, several diffraction peaks located at 30.54°, 35.99°, 43.76°, 57.91° and 63.62° corresponded to the (220), (311), (400), (511) and (440) planes of MnCo_2_O_4_ (PDF#23-1237). The change of these diffraction peaks confirmed the underlying transformation of the structure and the formation of spinel-phase-mixed metal oxide during calcination.

The FT-IR spectrum of CA, CoNiMn-CLDHs and CA-CoNiMn-CLDHs were employed to confirm the successful modification of CA on the surface of CoNiMn-CLDHs. As shown in Figure 2b, the broad band at 3454 cm^−1^ of CoNiMn-CLDHs was assigned to the O-H stretching vibration of surface hydroxyl groups and the adsorbed water on the sample surface. In the CA-CoNiMn-CLDHs, the peak at 3406 cm^−1^ represented the O-H stretching vibration of -OH, and the peak of 1721 cm^−1^ was consistent with the C=O symmetric stretching vibration in -COOH, while the peak observed at 1390 cm^−1^ could be ascribed to the asymmetric stretching vibration of C-O in -COOH [40,41]. The peak at 1629 cm^−1^ confirmed that the binding of CA molecules was mainly through the chemisorption between carboxylate citrate ions and CoNiMn-CLDHs. In addition, the peaks at 1205 and 1085 cm^−1^ were assigned to the C-O and C-H stretching vibrations, respectively, indicating the successful modification of CA on the CoNiMn-CLDHs surface [42].

The N_2_ adsorption–desorption isotherm of ZIF-67 and CA-CoNiMn-CLDHs was obtained to investigate the specific surface area and the pore size distribution. As displayed in Figure 2c, the isotherm of pure ZIF-67 was assigned as the typical type I with a small hysteresis loop according to the IUPAC classification, revealing the existence of abundant micropores [43,44]. The rapid increase in N_2_ adsorption capacity at a lower relative pressure (P/P_0_ < 0.5) also confirmed the presence of the micropores structure. As displayed in Figure 2d, the adsorption and desorption curves of CA-CoNiMn-CLDHs did not coincide completely and exhibited type-IV isotherms with a distinct H3 hysteresis loop, indicating the capillary condensation owing to the presence of the mesoporous. The microporous ZIF-67 was gradually etched into the hollow mesoporous LDH-based polyhedron with the replacement of anions in the 2-MIM ligand and the longitudinal growth of hydroxide monomers during the solvothermal reaction. Thus, the difference in pore structures between ZIF-67 and CA-CoNiMn-CLDHs might be related to the interlaced growth of ultrathin nanosheets on the facet of ZIF-67 template. The specific surface area was calculated using Brunauer–Emmett–Teller models (S_BET_), and the S_BET_ of CA-CoNiMn-CLDHs was 181.61 m^2^g^−1^, which was considerably smaller than that of ZIF-67 (892.32 m^2^g^−1^) (Table 2). The main reason for the decrease in specific surface area might be attributed to the disappearance of micropore structures of ZIF-67 by etching. Meanwhile, the hierarchical polyhedral, assembled by LDH nanosheets, formed larger ion-accessible mesopores, which were more favorable for efficient ion diffusion. Moreover, the pore size distributions of the samples were obtained by the Barrett–Joyner–Halenda mode (BJH) method, and the pore size of ZIF-67 and CA-CoNiMn-CLDHs were mainly concentrated in the range of 0–5 nm and 5–20 nm, respectively. The average pore diameter (D_p_) of CA-CoNiMn-CLDHs was 8.41 nm, exhibiting that the CA-CoNiMn-CLDHs mainly contained mesopores. The slit-like mesopores on the catalyst surface were conducive to the exposure of active sites, the diffusion of substrate and the improvement of hydrophilicity [45].

**Figure 3:**
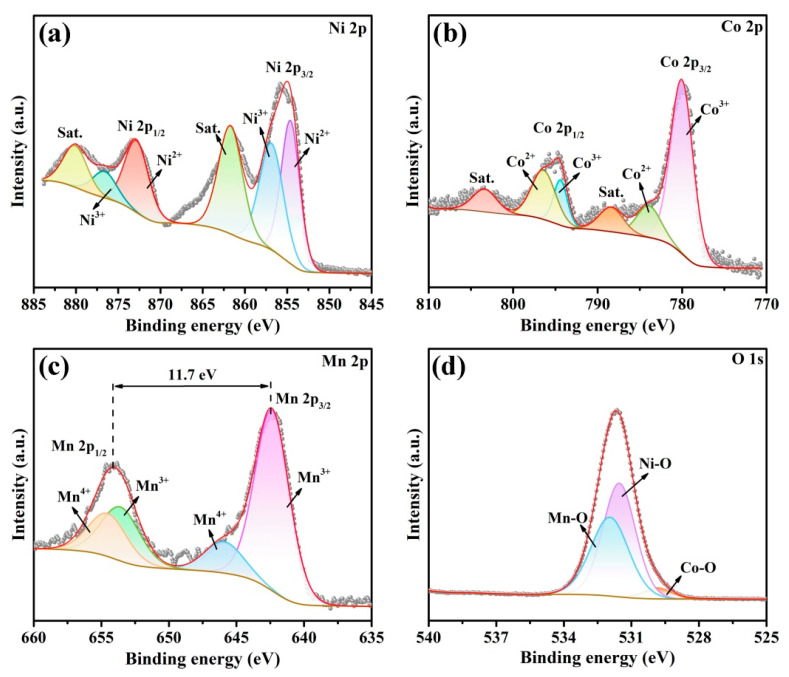
(**a**) Ni 2p, (**b**) Co 2p, (**c**) Mn 2p and (**d**) O 1s high-resolution XPS spectra of CA-CoNiMn-CLDHs.

In order to investigate the chemical composition and elemental chemical state of the CA-CoNiMn-CLDHs, XPS studies were conducted and the obtained XPS spectra were shown in Figure 3. As displayed in Figure 3a, the peaks centered at 854.61 and 872.82 eV were assigned to Ni^2+^, whereas the peaks with binding energies of 856.86 and 876.61 eV were attributed to Ni^3+^. In addition, the peaks located at 861.72 and 880.08 eV were ascribed to the vibrating satellites [46,47]. As illustrated in Figure 3b, the mixed Co^3+^ and Co^2+^ ions were found with the two peaks at 780.00 and 794.39 eV and corresponded to Co^3+^, and the peaks with binding energies of 783.93 and 796.40 eV were attributed to Co^2+^, while the peaks at 788.41 and 803.37 eV were typical Co vibrating satellites [48,49,50]. As depicted in Figure 3c, the Mn 2p spectra showed two peaks at 642.21 and 653.91 eV, which corresponded to the spin-orbit split of Mn 2p_3/2_ and Mn 2p_1/2_. Furthermore, the two peaks at 642.36 and 653.57 eV corresponded to Mn^3+^, and the two peaks centered at 645.91 and 654.52 eV were assigned to Mn^4+^ [48,51]. As an intermediate ion, Co^3+^ ion and Mn^3+^ ion could significantly promote the redox cycle during the catalytic process due to their strong conversion ability between various valence states of Co and Mn ions [52,53]. As shown in Figure 3d, the O 1s spectrum was fitted into three peaks located at 529.84, 531.54, and 531.95 eV, which were assigned to Co-O, Ni-O and Mn-O, respectively [54]. Therefore, the ZIF-67-derived CA-CoNiMn-CLDHs were successfully fabricated.

### 3.2. Peroxidase-like Activity and Mechanism of CA-CoNiMn-CLDHs

**Figure 4:**
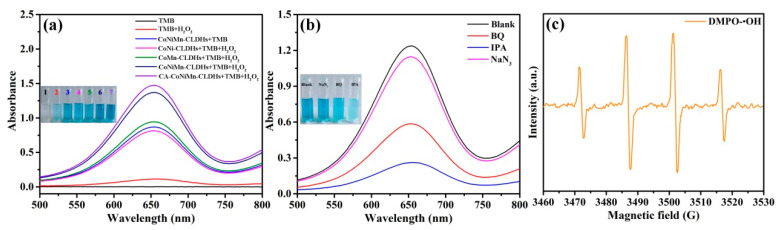
(**a**) UV-Vis spectra of different chromogenic systems. (**b**) Effect of scavengers on the absorbance of chromogenic system. (**c**) EPR spectra of DMPO-•OH obtained from the CA-CoNiMn-CLDHs/H_2_O_2_ reaction system.

To evaluate the peroxidase-like activity of CA-CoNiMn-CLDHs, 3,3′,5,5 tetramethylbenzidine dihydrochloride (TMB) was used as the typical choromogenic substrate for catalysis in the presence of H_2_O_2_. The comparison results for different reaction systems are shown in Figure 4a. The TMB and TMB+H_2_O_2_ reaction systems had no obvious absorption peak at 652 nm and no visible color change. Upon the addition of catalyst (CoNi-CLDHs, CoMn-CLDHs and CoNiMn-CLDHs) to the TMB+H_2_O_2_ reaction system, the colorless TMB transformed into blue oxTMB and a significant absorption peak appeared at 652 nm in the UV-Vis spectra, indicating that the catalyst had excellent peroxidase-like activity, while the CoNiMn-CLDHs+TMB reaction system also showed the distinct color change, revealing that the CoNiMn-CLDHs could directly oxidize TMB to produce oxTMB without the assistance of H_2_O_2_, which confirmed that CoNiMn-CLDHs also had oxidase-like activity. It is worth noting that the absorption peak intensity at 652 nm of the CA-CoNiMn-CLDHs+TMB+H_2_O_2_ system was remarkably enhanced, which was much higher than that of other reaction systems under the same condition, indicating that the introduction of CA notably enhanced the peroxidase-like activity of CA-CoNiMn-CLDHs. The surface of CA-CoNiMn-CLDHs was negatively charged due to the CA modification, while the TMB molecule with two amino groups was positively charged, thus accelerating the contact between TMB molecules and the catalytic sites on the CA-CoNiMn-CLDHs surface by the electrostatic interactions [55]. Therefore, CA-CoNiMn-CLDHs possessed a higher catalytic performance toward TMB in the presence of H_2_O_2_ than other control samples.

The catalytic mechanism of CA-CoNiMn-CLDHs was explored by the reactive oxygen species (ROS) capture assay. IPA, BQ and NaN_3_ were added into the chromogenic system (CA-CoNiMn-CLDHs+TMB+H_2_O_2_) to inhibit the production of •OH, O_2_^•−^ and ^1^O_2_, respectively. As illustrated in Figure 4b, the color change of the chromogenic system was inhibited and the absorbance at 652 nm dramatically dropped with the addition of IPA, indicating that •OH played a major part in the chromogenic reaction. When BQ was introduced into the chromogenic system, the absorption peak intensity at 652 nm also showed a trend of decline. This confirmed that a small number of O_2_^•−^ was produced in the reaction system. In contrast, upon the addition of NaN_3_, there was no obvious decrease in absorbance of the chromogenic system, indicating that ^1^O_2_ was not generated during the catalytic process. The corresponding ionic reaction equations are exhibited as follows. The dodecahedral CA-CoNiMn-CLDHs possessed Co^2+^/Co^3+^, Ni^2+^/Ni^3+^ and Mn^3+^/Mn^4+^ redox couples, which could decompose H_2_O_2_ into •OH through electron transfer under acidic conditions (Equation (1)). Simultaneously, Co^2+^, Ni^2+^ and Mn^3+^ could transfer electrons to the dissolved O_2_ and form O_2_^•−^ (Equation (2)). Previous studies showed that O_2_^•−^ could combine with H^+^ to produce H_2_O_2_ under acidic conditions, which was eventually transformed into •OH (Equations (1) and (3)). Therefore, •OH as the main ROS would oxidize TMB to oxTMB (Equation (4)) [56]. To further confirm the formation of •OH, the electron paramagnetic resonance (EPR) method was employed to characterize the •OH used by selecting the 5,5-dimethyl-1-pyr-roline N-oxide (DMPO) as the spin-trapping agent. As shown in Figure 4c, the introduction of CA-CoNiMn-CLDHs was promoted to produce characteristic DMPO-•OH peaks with an intensity ratio of 1:2:2:1, demonstrating that a large quantity of •OH was generated during the catalytic reaction. Therefore, the Co^2+^/Co^3+^, Ni^2+^/Ni^3+^ and Mn^3+^/Mn^4+^ redox pairs in CA-CoNiMn-CLDHs acted as electron mediators between oxidizing agents (O_2_ and H_2_O_2_) and the reducing agent (TMB) to realize the catalytic oxidation of TMB. Besides the ROS, Co^3+^, Ni^3+^ and Mn^4+^ could obtain electrons from the absorbed TMB and be reduced back to Co^2+^, Ni^2+^ and Mn^3+^, meanwhile, the colorless TMB would be oxidized into blue oxTMB (Equation (5)) [57].

(1) $${\mathrm{Co}}^{2+}/{\mathrm{Ni}}^{2+}/{\mathrm{Mn}}^{3+}+H_{2}O_{2}+H^{+}\overset{}{\to }{\mathrm{Co}}^{3+}{/\mathrm{Ni}}^{3+}/{\mathrm{Mn}}^{4+}+•\mathrm{OH}+H_{2}O$$

(2) $${\mathrm{Co}}^{2+}/{\mathrm{Ni}}^{2+}/{\mathrm{Mn}}^{3+}+O_{2}\overset{}{\to }{\mathrm{Co}}^{3+}/{\mathrm{Ni}}^{3+}/{\mathrm{Mn}}^{4+}+O_{2}^{•-}$$

(3) $$O_{2}^{•-}+2H^{+}\overset{}{\to }H_{2}O_{2}+O_{2}$$

(4) $$•\mathrm{OH}+\mathrm{TMB}\overset{}{\to }\mathrm{oxTMB}+H_{2}O$$

(5) $${\mathrm{Co}}^{3+}{/\mathrm{Ni}}^{3+}/{\mathrm{Mn}}^{4+}+\mathrm{TMB}\overset{}{\to }{\mathrm{Co}}^{2+}{/\mathrm{Ni}}^{2+}/{\mathrm{Mn}}^{3+}+\mathrm{oxTMB}$$

**Figure 5:**
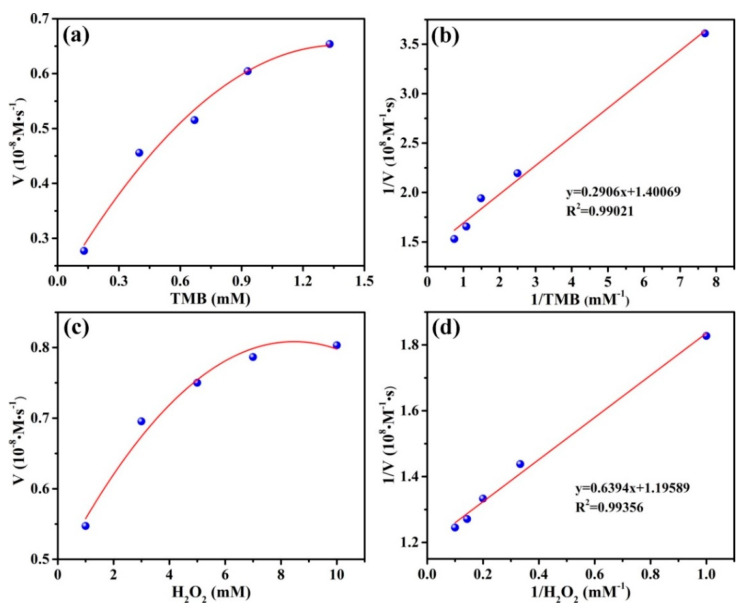
(**a**) Michaelis–Menten plot and (**b**) corresponding Lineweaver–Burk plot for variation of TMB. (**c**) Michaelis–Menten plot and (**d**) corresponding Lineweaver–Burk plot for variation of H_2_O_2_.

The steady-state kinetic assay was carried out to further evaluate the catalytic activity of CA-CoNiMn-CLDHs with TMB and H_2_O_2_ as the targeted substrates. The kinetic parameters K_m_ and V_max_ were obtained by using the Lineweaver–Burk plots through Michaelis–Menten equation (Equation (6)):

(6) $$\frac{1}{v}=\frac{1}{\left[S\right]}\times \frac{K_{m}}{V_{\max}}+\frac{1}{V_{\max}}$$

where v stands for the initial velocity, [S] is the concentration of TMB and H_2_O_2_, V_max_ is the maximal reaction velocity, and K_m_ is the Michaelis–Menten constant. Among them, K_m_ was an effective parameter to evaluate the affinity between CA-CoNiMn-CLDHs and peroxidase substrates (TMB and H_2_O_2_). The typical Michaelis–Menten curves and Lineweaver–Burk plots are displayed in Figure 5. Table 3 summarizes the K_m_ and V_max_ of CA-CoNiMn-CLDHs in comparison to the nature of horseradish peroxidase (HRP). The K_m_ of CA-CoNiMn-CLDHs was 0.21 mM for TMB and 0.53 mM for H_2_O_2_, respectively, which were lower than that of HRP to TMB (0.43 mM) and H_2_O_2_ (3.7 mM), demonstrating that a strong affinity between CA-CoNiMn-CLDHs and each substrate. The high affinity for substrate might benefit the subsequent catalytic reactions and could realize sensitive determination of trace contaminants in water.

### 3.3. Detection of Phenol by CA-CoNiMn-CLDHs

**Figure 6:**
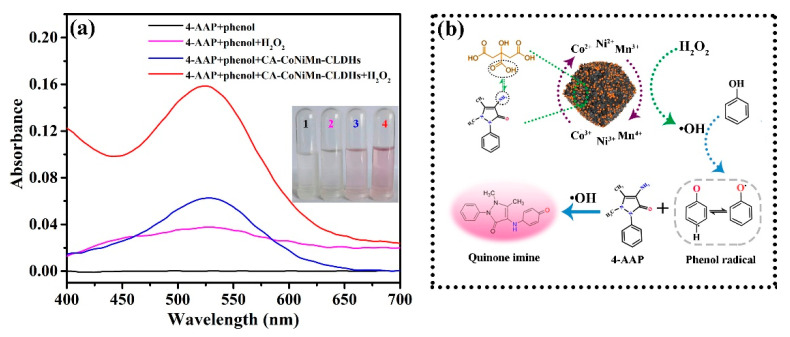
(**a**) UV-Vis spectra of different reaction systems using 4-AAP as the substrate. (**b**) Possible detection mechanism of the CA-CoNiMn-CLDHs-base colorimetric detection platform.

Based on the excellent peroxidase-like activity of CA-CoNiMn-CLDHs, a facile and rapid colorimetric sensing platform for phenol was built. The decomposition of H_2_O_2_ by CA-CoNiMn-CLDHs produced •OH, which could induce the oxidative coupling of phenol and 4-AAP to form a pink-quinoid dye with a characteristic absorption peak at 525 nm [58]. As depicted in Figure 6a, the combination of phenol and 4-AAP had no characteristic absorption peak at 525 nm, while the system with the addition of H_2_O_2_ produced a pale pink. The reaction system also showed a weak pink color after the addition of CA-CoNiMn-CLDHs due to the production of a small amount of O_2_^•−^. By comparison, the reaction system with the coexistence of CA-CoNiMn-CLDHs and H_2_O_2_ produced a noticeable color change from colorless to distinct pink, and exhibited a dramatic increase in the characteristic absorption at 525 nm. The above results indicated that CA-CoNiMn-CLDHs showed the most excellent catalytic performance, which could be ascribed to the synergistic effect of Co, Ni and Mn ions and the modification of CA. As the typical transition metal element, Co, Ni and Mn had multiple valence states and the Co^2+^, Ni^2+^ and Mn^3+^ showed peroxidase-like activity. Additionally, the synergistic effect between Co, Ni and Mn would bring new active sites for the catalytic reaction and participate in the generation of more •OH [59]. The possible reaction mechanism for the oxidative coupling of phenol and 4-AAP by •OH was shown in Figure 6b. The CA-CoNiMn-CLDHs with peroxidase-like activity could induce the O-O bond breakage of H_2_O_2_ to produce strong oxidizing •OH. The •OH would grab the single electron from the phenolic hydroxyl group to form the quinoid radicals, and then the excess •OH triggered the oxidative coupling reaction of quinoid radicals and 4-AAP to generate a pink-quinoid dye [60]. In addition, the carboxyl group (-COOH) in citric acid combined with the amino group (-NH_2_) in 4-AAP through hydrogen bonding (N-H-O), which accelerated the adsorption of substrate molecules (4-AAP) on the catalyst surface. Simultaneously, the oxidative coupling reaction between phenol and the absorbed 4-AAP was triggered rapidly by the •OH produced on the catalyst surface. Therefore, CA functionalization contributed to improving the catalytic activity of the as-prepared catalyst.

The peroxidase-like activity of CA-CoNiMn-CLDHs was affected by pH, reaction time, incubation temperature and reactant concentration (4-AAP, H_2_O_2_ and CA-CoNiMn-CLDHs).

**Figure 7:**
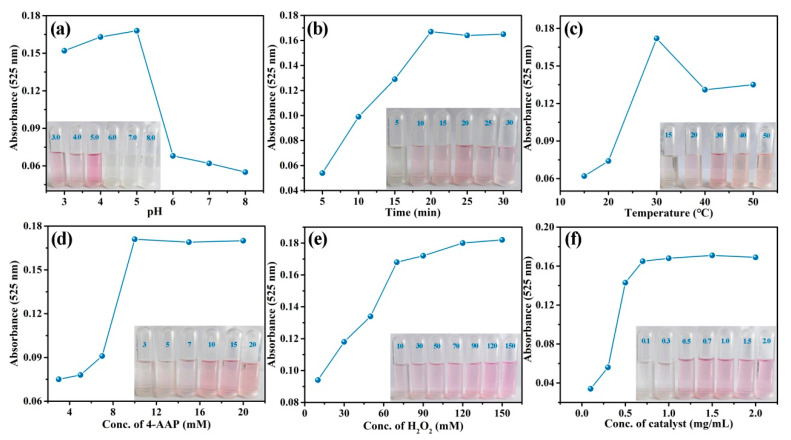
Effect of (**a**) pH, (**b**) incubate time, (**c**) temperature, (**d**) TMB concentration, (**e**) H_2_O_2_ concentration and (**f**) CA-CoNiMn-CLDHs concentration on the chromogenic system.

In order to obtain the optimum catalytic conditions, the optimization experiment was carried out and the concerning results are shown in Figure 7. Figure 7a shows that the color of the reaction system prominently changed from colorless to pink at pH 5.0 and the absorbance at 525 nm was the highest, indicating the catalytic activity of CA-CoNiMn-CLDHs reached the maximum under the weak acid environment. Therefore, pH 5.0 was used as the optimal pH for the detection of phenol in the subsequent experiments. Figure 7b suggests that the absorbance of the reaction system increased with the reaction time and reached a platform at 20 min. Figure 7c displays the influence of the incubation temperature (15–50 °C) on the oxidative coupling reaction, and the absorbance of the reaction system gradually increased from 15 to 30 °C, while it decreased with the further increase of temperature, which might be attributed to the inactivation of the catalyst and rapid decomposition of H_2_O_2_ at a higher temperature. Hence, the optimal temperature was 30 °C. Moreover, the absorbance of reaction system was closely related to the concentration of 4-AAP, H_2_O_2_ and CA-CoNiMn-CLDHs. As shown in Figure 7d–f, the absorbance at 525 nm increased rapidly with the increase of the reagents’ (4-AAP, H_2_O_2_ and CA-CoNiMn-CLDHs) concentrations. When the concentration of reagents increased to a certain extent, the absorbance no longer increased significantly. Therefore, the optimum concentrations of 4-AAP, H_2_O_2_ and CA-CoNiMn-CLDHs were 10 mM, 70 mM and 0.7 mg/mL, respectively.

**Figure 8:**
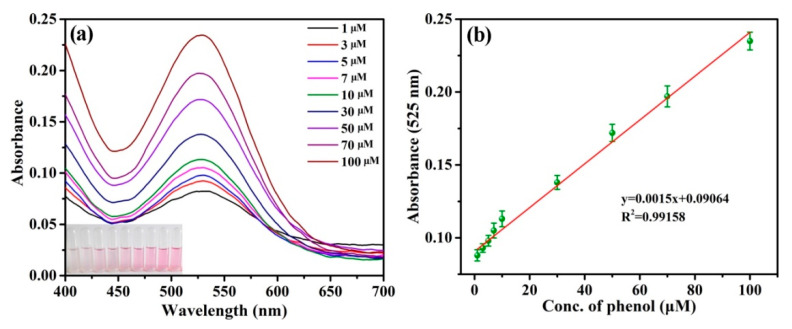
(**a**) UV-Vis spectra of the chromogenic system with the addition of different concentration of phenol. (**b**) The linear relationship between the absorbance and phenol concentration.

Under the optimal experimental conditions, colorimetric determination for phenol was carried out to obtain the linear relationship between phenol concentration and absorbance at 525 nm. As depicted in Figure 8a,b, the UV-Vis absorbance increased with the increase of phenol concentration from 1 to 100 μM, which presented an excellent linearity between phenol concentrations and absorbance (R^2^ = 0.99158). The limit of detection (LOD) for phenol was calculated as 0.163 μM, according to the 3σ rule (3σ/slope). Furthermore, in comparison with the reported methods for phenol determination, the colorimetric sensor platform base on the CA-CoNiMn-CLDHs had higher detection sensitivity and efficiency (Table 4). This was because the hierarchical hollow CA-CoNiMn-CLDHs possessed the characteristics of a higher specific surface area, enhanced active site, abundant pore structures and shorter electron transport distance, which could promote the combination of catalyst and substrate, thus increasing the mass transfer efficiency, accelerating the reaction rate, and showing excellent catalytic performance.

### 3.4. Evaluation of Selectivity, Reusability and Stability of CA-CoNiMn-CLDHs

**Figure 9:**
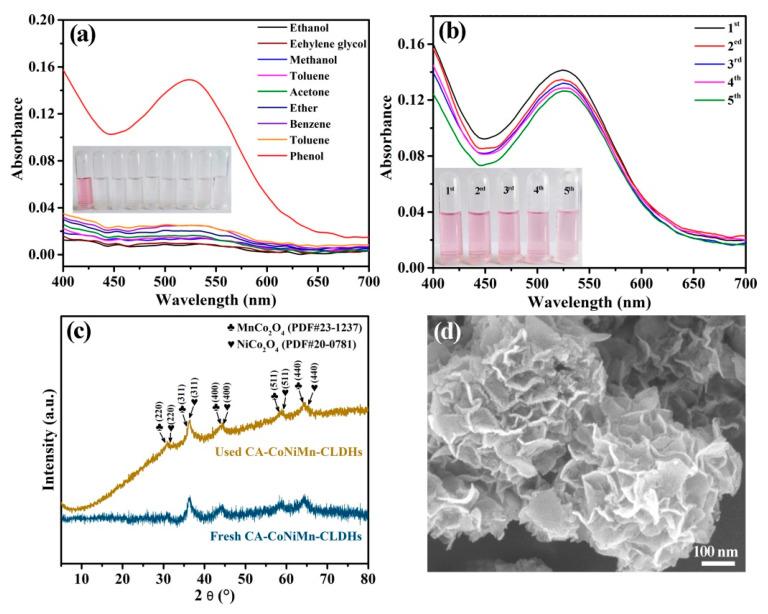
(**a**) Selectivity of the chromogenic system for phenol compared to other interfering substances. (**b**) Reusability of CA-CoNiMn-CLDHs for phenol colorimetric detection. (**c**) XRD patterns of CA-CoNiMn-CLDHs before and after detection of phenol. (**d**) SEM image of CA-CoNiMn-CLDHs after use.

The good selectivity and high-cycling stability of the sensors were essential factors for expanding the application and reducing the usage cost. Firstly, the selectivity of the designed colorimetric sensing platform was further investigated by comparing the absorbance change with the addition of phenol and other common organic interfering compounds (ethanol, methanol, cyclohexane, ether, benzene, acetonitrile, acetone and toluene). As shown in Figure 9a, only the reaction system with the addition of phenol exhibited rapid color change and generated a pink-quinoid-type dye that showed absorbance maxima at 525 nm, while the control groups containing the interfering substances had no obvious absorption peak at 525 nm. The above results indicate that the interference of these organic compounds for phenol detection could be ignored, and the established colorimetric sensing platform possesses high selectivity and anti-interference capacity. In addition, the reusability and stability of CA-CoNiMn-CLDHs were studied through a cycle experiment and the result is presented in Figure 9b. CA-CoNiMn-CLDHs displayed excellent cycling stability after five cycles. The peroxidase-like catalytic activity had no significant decrease and still retained 82.6% of its original activity. Notably, the slight decrease in catalytic activity might be ascribed to the coverage of partial active sites and the loss of the catalyst during the recovery process. The XRD pattern and the SEM image of CA-CoNiMn-CLDHs after use are depicted in Figure 9c,d; the main characteristic diffraction peaks of CA-CoNiMn-CLDHs still existed and the original morphology of CA-CoNiMn-CLDHs basically remained, revealing that the as-prepared catalyst had high chemical and structural stability in the process of phenol detection. Therefore, the synthesized CA-CoNiMn-CLDHs were a promising nanozyme for the detection of phenol.

## 4. Conclusions

In summary, ZIF-67-derived, hollow CA-CoNiMn-CLDHs with excellent peroxidase-like activity were synthesized through a facile solvothermal reaction. The synthesized CA-CoNiMn-CLDHs displayed a multi-order hollow micro-nanostructure, which was assembled by ultrathin nanosheets that grew interlaced on the surface of the ZIF-67 precursor. The highly exposed active sites and abundant pore structures facilitated the rapid electron transport and enhanced the synergy between nickel, cobalt and manganese ions, thereby efficaciously boosting the peroxidase-like catalytic activity of CA-CoNiMn-CLDHs. Additionally, the CA modification further improved the affinity of the as-syntheized catalyst to the substrates. The CA-CoNiMn-CLDHs-mediated colorimetric detection platform was successfully applied for the sensitive visual detection of phenol in water with a detection limit of 0.163 μM. Additionally, the chemical structure and catalytic activity of CA-CoNiMn-CLDHs after use demonstrated no considerable change, indicating that the as-synthesized catalyst had higher stability and reusability. Thus, the design concept of the ZIF-67-derived, three-dimensional hollow nanozyme reported in this paper provides a facile strategy for the developing of a novel MOF-derived nanozyme for the detection of contaminants in polluted water.

## Figures and Tables

**Table 1:** Synthesis conditions for the preparation of CoNi-LDHs and CoMn-LDHs.

Sample	Ni(NO_3_)_2_·6H_2_O (mg)	MnCl_2_·4H_2_O (mg)	Solvent
CoNi-LDHs	160	0	DMF/EtOH/DIW
CoMn-LDHs	0	160	DMF/EtOH/DIW

**Table 2:** Pore texture parameters of as-syntheized samples.

Sample	*S*_BET_ (m^2^g^−1^)	*V*_Total_ (cm^3^g^−1^)	*D*_p_ (nm)
ZIF-67	892.32	0.83	2.02
CA-CoNiMn-CLDHs	181.61	0.52	8.41

**Table 3:** Comparison of the kinetic parameters of CA-CoNiMn-CLDHs and HRP.

Materials	K_m_ (mM)		V_max_ (10^−8^ M s^−1^)	
	H_2_O_2_	TMB	H_2_O_2_	TMB
HRP	3.7	0.43	8.71	10
CA-CoNiMn-CLDHs	0.53	0.21	0.71	0.83

**Table 4:** Comparison of various catalysts for the detection of phenol.

System	LOD (μM)	Linear Range	Ref.
CAT-Fe_3_O_4_@ZIF-8	0.7	5–100 μM	[1]
Co_3_O_4_/CDM	1.02	0.5–300 μM	[3]
Au@Ni13/rGO	1.68	1–300 μM	[8]
Co/Mn-MOF-74	0.2	0.5–500 μM	[59]
MoS_2_-Pt_3_Au_1_	0.2	4–1000 μM	[61]
N, Cu-CQDs	0.12	1–100 μM	[60]
HRP-Cu_3_(PO_4_)_2_·3H_2_O	1	0–100 μM	[62]
CA-CoNiMn-CLDHs	0.163	1–100 μM	This work

## Data Availability

Data is contained within the article.
